# INTEGRATOR: interactive graphical search of large protein interactomes over the Web

**DOI:** 10.1186/1471-2105-7-146

**Published:** 2006-03-16

**Authors:** Aaron N Chang, Jason McDermott, Zachary Frazier, Michal Guerquin, Ram Samudrala

**Affiliations:** 1 Dept. of Biomedical and Health Informatics, University of Washington, Seattle, Washington 98195, USA; 2 Dept. of Microbiology, University of Washington, Seattle, Washington 98195, USA; 3 Rosetta Inpharmatics LLC, A wholly owned subsidiary of Merck & Co. Inc., Seattle, Washington 98109, USA

## Abstract

**Background:**

The rapid growth of protein interactome data has elevated the necessity and importance of network analysis tools. However, unlike pure text data, network search spaces are of exponential complexity. This poses special challenges for storing, searching, and navigating this data efficiently. Moreover, development of effective web interfaces has been difficult.

**Results:**

We present Integrator, a web-integrated graphical search tool for protein-protein interaction networks across 50+ genomes.

**Conclusion:**

Integrator provides single and multiple protein searches of the Bioverse database containing experimentally-derived and predicted protein-protein interactions. The interface provides animated local network views, rapid subgraph manipulation, and cross-referencing of functional annotations. Integrator is available at .

## Background

High-throughput technologies that monitor cellular components on a large scale are becoming ubiquitous in the post-genomic era. An important analytical paradigm in systems biology is the molecular interaction network [1]. Networks provide an intuitive visualization of component relationships and are amenable to quantitative graph analysis. Constituents include genes, proteins, small molecules or combinations thereof [2-5]. In particular, public repositories of protein-protein interaction (PPI) data collected from yeast two-hybrid arrays, affinity chromatography, and manual curation methods have grown significantly in recent years [6-8].

Building search tools that effectively navigate these interaction networks remains a significant informatics challenge. This is due in large part to the exponential complexity of the search space, rapid data turnover, decentralized storage of primary data, and diversity in data models [9]. Taking this into consideration, we present Integrator, a tool for the analysis of PPI networks using a centralized data model. Integrator is composed of a highly interactive, low-memory overhead network viewer with an enterprise-level application server back-end accessing data from the Bioverse project [10,11]. This database contains a large collection of experimentally-derived and predicted PPI data for over 50 genomes based in part by applying the Interolog prediction method [12]. Interologs are interactions predicted between proteins in one species using experimental interaction evidence and the relative sequence homologies to proteins in an orthologous species. Such predictions have been used to extrapolate novel functional annotations for previously unannotated proteins with high accuracy [13].

In contrast to stand-alone network viewer applications, including one previously released by our group [14], the Integrator interface is completely intertwined with a server-based web application. This means several million PPIs stored in a relational database can be explored quickly over the web through common web browsers with minimal additional software. Integrator provides several new graph manipulation features that significantly improve upon tools previously released. It also performs multiple protein searches where protein identifier sets can be compared or contrasted by connected graph components. Integrator is a simple, all-in-one graphical search solution for large interactomes across several genomes.

## Implementation

Integrator is based on a three-tier web application architecture using the Java-based Struts web application framework [15]. This design partitions the client, server, and database into three separate information layers. Advantages to this approach include the avoidance of having users install memory-intensive client programs, especially whenever an upgrade becomes available [16] and placing computational load away from clients and onto high-performance servers. The Struts model-view-controller (MVC) paradigm is used to organize tasks including node and edge searches, identifier synonym resolution, viewer assembly, and database query. The JUNG graph analysis Java library is used for connected component analysis in multiple protein searches [17]. The network viewer is a modification of the Touchgraph Java applet viewer [18]. The data layer contains non-redundant pairwise PPI data (experimentally derived and predicted) from the Bioverse project warehoused in a MySQL database as described previously [10,11].

## Results and discussion

### Single protein identifier search

To search networks around a specific protein, Integrator first attempts to locate exact or similar identifier matches to a given query. If a single match is found, the user is returned a graph around the query protein. If similar identifier matches exist, the user is given a list from which to narrow the search. Links to sequence and functional annotation data for each protein are provided to aid this process. Integrator currently recognizes a number of identifiers including those from Genbank, Flybase, Wormbase, and the Saccharomyces Genome Database [10,19-23].

**Figure 1:**
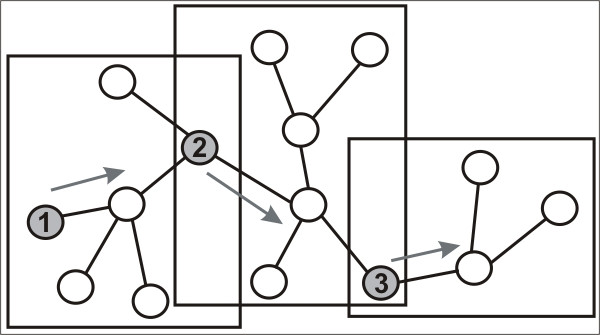
Frame-by-frame network navigation. A search begins at node 1 in the left-most window. Once node 2 is reached in the middle window, a new search is performed to center the network around that node. This process is repeated at node 3 in the right-most window. A connected network can be fully traversed using this method. This localized navigation approach works well to reconcile the exponential complexity of networks and the limitations of two-dimensional viewers.

To provide a visual interface for traversing network results, we implemented an interactive, frame-by-frame navigation solution (Figure 1). A network neighborhood (depth = 3) around a query protein is initially generated by a breadth-first search. Within this network, a user can interactively expand, contract, add, or subtract nodes and edges from the viewer. This allows for dynamic manipulation of network components to aid visual analysis. When a user wishes to expand the network in greater detail around a specific node, contextual menus (right-click on nodes) can be used to re-center the graph around it. By repeating this process, a user can explore an entire connected network starting from any node.

**Figure 2:**
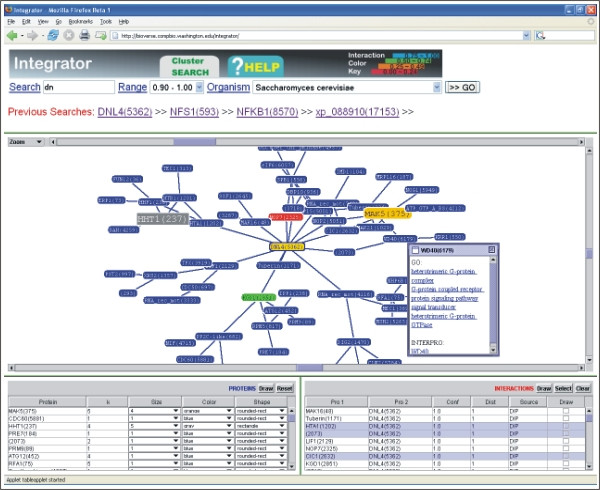
The Integrator network viewer interface. Shown here is a representative network search result around a selected node (yellow) with various other nodes modified for color, size, or shape. The center frame contains the main interactive graph viewer. Users can traverse a network by clicking on nodes. Double-clicking on a node opens a window containing detailed sequence and annotation information. Hovering over nodes also brings up tool tips containing GO and Interpro annotations. Hovering over edges shows tool tips with edge confidence data. Right-click contextual menus also exist for nodes and edges which allow for search, hiding, showing, and changing their visual properties. The slider bar at the top of the graph viewer modulates zoom, rotation, or viewable neighborhood size. Below this are two interactive network tables, nodes on the left, edges on the right. These tables can be used to sort and modify various node and edge properties in the viewer.

Networks are represented in graph and table formats in the client browser (Figure 2). The graph viewer is based on the open-source Touchgraph viewer which provides several built-in features like pan, zoom, rotation, neighborhood view adjustment, and tool tips [18]. Graph components consist of proteins as nodes and undirected edges between them for physical interactions. The edges are color-coded by confidence value as assessed by the Bioverse project. The nodes are labeled with gene symbol identifiers or Interpro functional annotations, depending on availability [23]. These identifiers are also suffixed with unique Bioverse ID numbers to distinguish between potential isoforms or splice variants. Hovering over a node yields a tool tip containing hyperlinked functional annotations from the Gene Ontology (GO) and Interpro classification systems [23,24]. Edge tool tips contain database source information and confidence values.

Two tables are also provided below the main viewer, which list the proteins and their interactions. The protein (node) table can be used to sort and manipulate node size, shape, or color. Similarly, the interaction (edge) table can be used to sort columns and select specific interactions to render subgraphs in the graph window. A detailed navigation tutorial is provided online.

### Multiple protein identifier search

**Figure 3:**
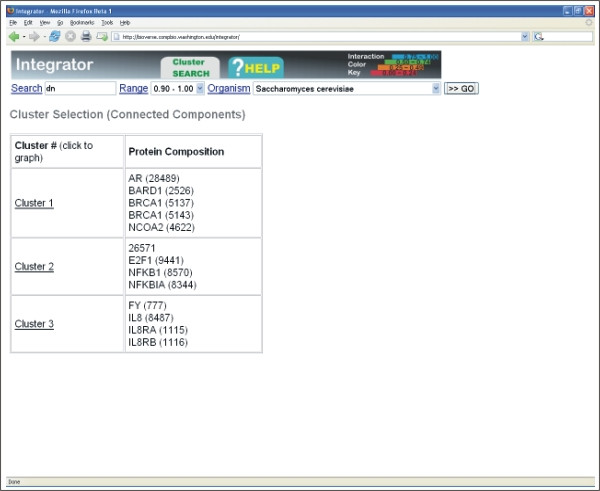
Multiple protein identifier search results. A representative search result where three clusters (defined by connected graph components) are shown with their constituent nodes. Each of the individual clusters can be viewed graphically by following their hyperlinks.

Integrator also provides the option to search multiple protein identifiers simultaneously. These batch searches are constrained to direct PPIs only (depth = 1). The resulting network is used to determine connected component profiles, or unbroken edge clusters, among them (Figure 3). Each individual cluster is then made available for display using the graphical interface. Additionally, users can compare or contrast PPIs between two different sets of proteins.

## Conclusion

Integrator provides a simple, unified interface for interactome data using familiar web technology. The learning curve required is relatively low and installation of client software is minimal. As a result, users can focus quickly on network analysis. By restricting searches to varying ranges of PPI (edge) confidence values, a user can compare different networks for a given set of proteins. Users have the added flexibility of manipulating subgraphs within each network using graphical and tabular interfaces.

One restriction for users is the method of PPI prediction scoring currently employed in the Bioverse database that is based on the Interolog method [12]. However, users do have the option to restrict their analysis to original source PPIs by filtering for interactions with the highest confidence score (1.0) using the table interface. As new PPI data sets become available, we will continue to update the database, meaning search results are likely to change over time. Although the current database is limited to intra-genomic PPIs, efforts are underway to compile inter-genomic PPIs (i.e. host-pathogen PPIs).

Like other two-dimensional graph viewers, there is a practical upper-bound limit on the number of nodes in a network (~500) that can be effectively viewed at once. This is commonly due to spatial crowding produced by existing layout algorithms. Integrator attempts to overcome this limitation by utilizing multiple-frame searches. An alternative solution for viewing large global networks might be the use of three-dimensional hyperbolic layout viewers that can interactively display >100,000 simultaneous nodes [25]. Such viewers are promising approaches to network visualization with the caveat that they are dependent on well-defined minimum spanning trees and require significant computational overhead. Two-dimensional viewers will likely remain the preferred interface of choice for related, web-based search technologies.

**Figure 4:**
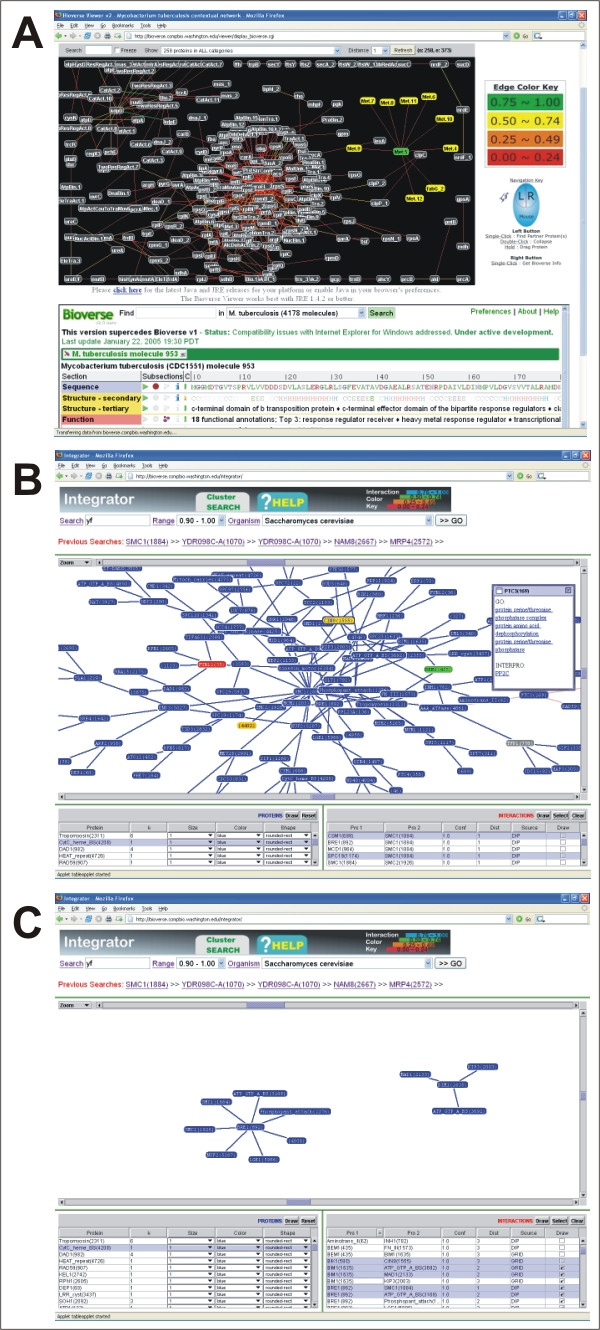
Comparison of a graph-only viewer versus the graph-plus-table interface as provided by Integrator. (A) Network viewer without a table interface, previously released by our group (). Subgraph operations are performed one node or edge at a time. (B) Integrator network before subgraphing. (C) Integrator network after subgraphing. Subgraphs can be specified on any number of node or edge properties just by sorting columns and selecting the desired rows.

A critical new feature in network navigation introduced by Integrator, with respect to other published network viewers, is the interactive table interface. Graph-centric viewers are powerful but generally lack the capacity to render subgraphs rapidly to specific nodes or edges. The table interface in Integrator remedies this shortcoming by allowing users to sort columns on various properties and subselect nodes and edges. To illustrate this point, we compared a graph-only viewer, previous released by our group, versus the Integrator interface (Figure 4). We found that graph-only viewers restricted subgraphing to a node-by-node or edge-by-edge operation, causing significant delay in analysis. The table interface within Integrator far exceeded the graph-only viewer in terms of speed and ease-of-use. The complex visual aspect of network analysis requires that these operations occur quickly, especially when users wish to filter networks against specific molecular criteria. In practice, decomposition of networks in this manner appeared to aid hypothesis generation for most end-users we have encountered.

Currently, our group is working towards providing an advanced text search solution through the Bioverse search homepage (). This will include significant enhancements in Boolean queries and complex query handling. We are working towards a tighter integration of this interface with Integrator's network analysis tools. Furthermore, by making the Integrator codebase available to the public, we hope that integration with similar or derivative projects will provide interesting new features in the future.

## Availability and requirements

Integrator is freely available to all users through any Java-enabled web browser at . All visits to the web application preserve user and data anonymity. A source code distribution is available at this site.

## Authors' contributions

ANC designed and implemented the Integrator web application. JM computed and updated the Bioverse dataset. ZF designed and implemented the Bioverse relational database. MG assisted with database design and database optimization. RS oversaw original design specifications, coordinated all relevant projects and assessed development milestones. All authors read and approved the final manuscript.
